# A New Entity of Ramp Lesion Combined with Posterior Root Tear of the Medial Meniscus: A Case Report

**DOI:** 10.2106/JBJS.CC.23.00439

**Published:** 2024-05-03

**Authors:** Andrea Di Muro, Zyad Ayman Taha, Jacopo Corti, Fabio Frasconà, Fabrizio Matassi

**Affiliations:** 1 Department of General Orthopedic, University of Florence, A.O.U. Careggi CTO, Florence, Italy

**Keywords:** ramp lesion, root tear, medial meniscus, arthroscopy, ACL revision, knee instability, knee surgery

## Abstract

**Case:**

This report describes a new pattern of meniscal tear in an 18-year-old man after a knee sprain; he had undergone anterior cruciate ligament revision (ACL-R) 3 years earlier. He was diagnosed with an anterior cruciate ligament (ACL) graft rupture, a ramp lesion (Thaunat type 4), and a posterior root avulsion fracture of the medial meniscus (MM) (LaPrade type 5). He was treated successfully with an all-inside repair of the ramp lesion, a transtibial pull-out repair of the root tear, and ACL graft revision and anterolateral stabilization.

**Conclusion:**

This specific meniscal injury pattern should be recognized and documented, potentially warranting consideration as a new addition to Thaunat and LaPrade classifications as type 6.

ACL injuries often involve associated lesions of the meniscus such as ramp lesions or posterior root tears. Ramp lesions are peripheral longitudinal tears of the posterior horn of the medial meniscus, involving the meniscocapsular ligament, the meniscotibial ligament, and/or the red-red zone of the posterior horn. They are classified into 5 types according to Thaunat et al^1^. Root tears, on the other hand, are bony or soft-tissue root avulsion injuries or radial tears within 1 cm of the root attachment of the meniscus (lateral or medial), and they are classified into 5 types by LaPrade et al.^2^ (Tables I and II).

**TABLE I:** Thaunat Ramp Lesions Classification

Type 1	Meniscocapsular lesions: These lesions are very peripherally located in the synovial sheath.
Type 2	Partial superior lesions: These lesions are stable and can be diagnosed only by a transnotch approach.
Type 3	Partial inferior or hidden lesions: The lesions are not visible with the transnotch approach but may be strongly suspected when there is significant mobility at probing.
Type 4	Complete tear in red-red zone. Mobility at probing is very high.
Type 5	Double tear lesions.

**TABLE II:** LaPrade Root Lesion Classification

Type 1	Partial stable tear.
Type 2	Complete radial tear within 9 mm from attachment.
Type 3	Bucket-handle tear with complete root detachment.
Type 4	Complex oblique or longitudinal tear with complete root detachment.
Type 5	Bony avulsion fracture of the root attachment.

These lesions are associated with increased anterior tibial translation, dynamic rotational laxity, and excessive rotational knee motion, thus leading to increased knee biomechanical instability^6^. Furthermore, meniscal root tears increase contact pressures and can lead to the articular cartilage damage and early osteoarthritis if not adequately treated. If any of these lesions are not identified and treated properly, they can lead to anterior cruciate ligament (ACL) reconstruction failure^10^. This makes it obligatory to thoroughly inspect the meniscus and treat any lesions accordingly, especially during ACL reconstruction or revision.

In this case report, we present a patient with ACL rerupture, who had a combined ramp lesion and bony avulsion of the posterior root of the medial meniscus (MM) of the left knee. The patient was treated with arthroscopic repair using an all-inside technique for the ramp lesion and a transtibial technique to repair the root lesions, along with ACL revision and anterolateral stabilization.

We did not find any reported cases in the scientific literature of ramp lesions associated with bony root avulsion affecting the same meniscus.

The patient and his parents were informed that data concerning the case would be submitted for publication, and they provided consent.

## Case Report

A 15-year-old boy was diagnosed with an ACL tear after sustaining an injury to the left knee while skiing. A few days after the trauma, he underwent arthroscopic ACL reconstruction using an ipsilateral hamstring autograft. During arthroscopy, no additional injuries beyond the ACL were detected. The patient had returned to normal-high physical activity levels 7 months after the surgery.

At 18 years, this patient presented to our clinic with persistent knee instability over 2 months after a knee injury during soccer practice. Physical examination revealed slight knee effusion, an active range of motion of 0-0-120° because of severe pain, and 6-10 mm of anterior tibial translation (Grade II) during the Lachman test, when compared with the uninjured side. A noticeable “clunk” associated with pain was observed during the pivot shift test. The McMurray test was positive for a lesion of the medial meniscus. The patient had pain on palpation of the medial joint line. The knee was stable during varus and valgus stress tests, as well as during the posterior drawer test.

MRI revealed a complete ACL graft rupture and a complex tear of the medial meniscus (ramp lesion and bony avulsion of the posterior root of the MM) (Fig. 1).

**Figs. 1-A and 1-B** In (**Fig. 1-A**) (T2 Turbo Spin Echo fast spin sag MRI) type 4B ramp lesion. Complete tear of the meniscotibial ligament at its attachment to the posterior horn of the medial meniscus. The T2 sequence showed fluid intensity signal extending from the superior to the inferior articular surface with disruption of the capsular ligaments. In (**Fig. 1-B**) (T1 Turbo Spin Echo fast spin sag MRI) type 5 posterior root lesion (with bone avulsion). Bony avulsion of posterior root of MM visible anterior to the Posterior Cruciate Ligaments.

**Fig. 1-A:**
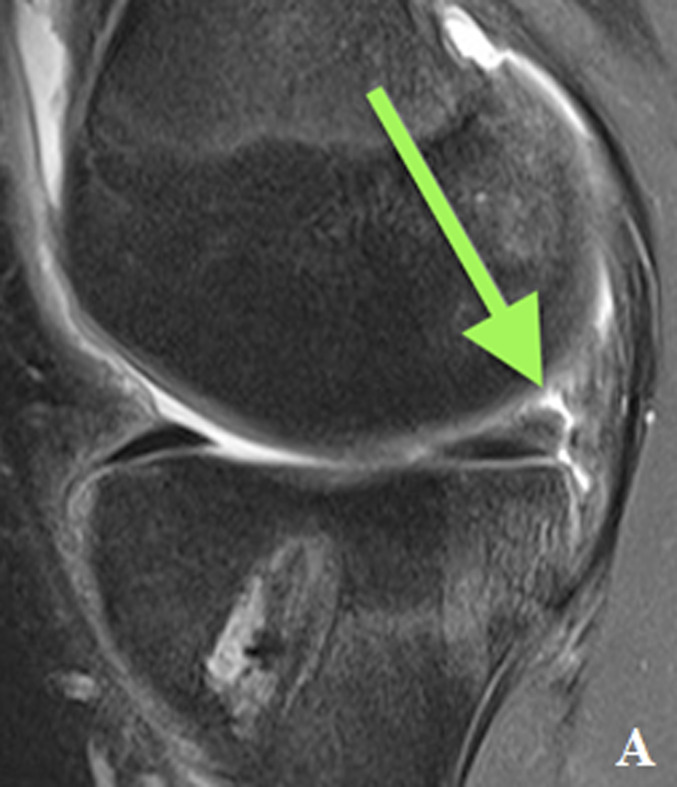

**Fig. 1-B:**
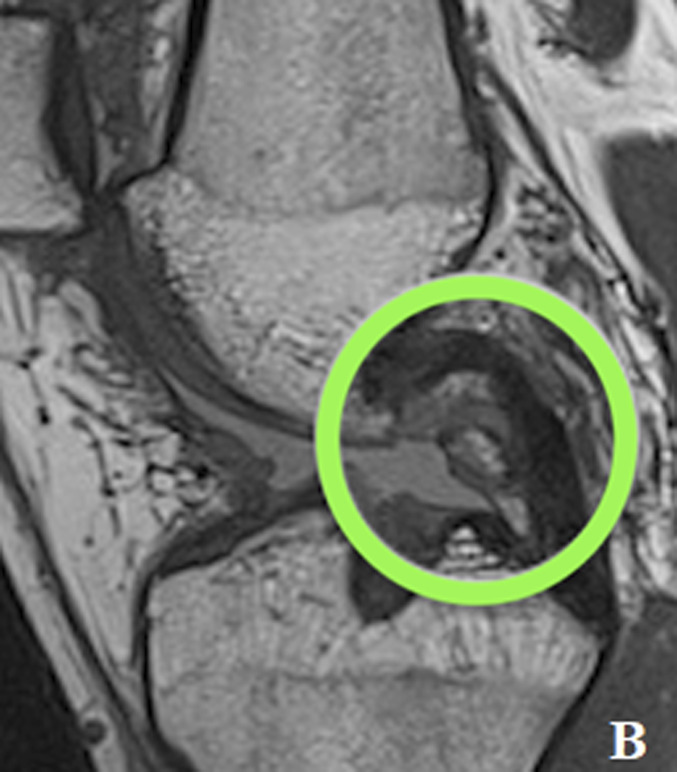

Preoperative knee CT was performed to plan the ACL revision and assess the position of the tunnels and any osteolysis. The CT showed no significant widening of both tunnels. According to Bernard and Hertel's grid^3^, the tibial tunnel was semianatomic, and the femoral tunnel was vertical-anterior in relation to the anatomic footprint (green dot in Fig. 2). The CT also revealed a bone fragment in the intercondylar notch, indicating bony avulsion of the posterior root of the MM (Fig. 3).

**Figs. 2-A and 2-B** Bernard and Hertel's grid on the CT scan showing vertical-anterior femoral tunnel (**Fig. 2-A**) and semianatomic tibial tunnel (**Fig. 2-B**). Green dots indicate the femoral (**Fig. 2-A**) and tibial (**Fig. 2-B**) anatomic footprint.

**Fig. 2-A:**
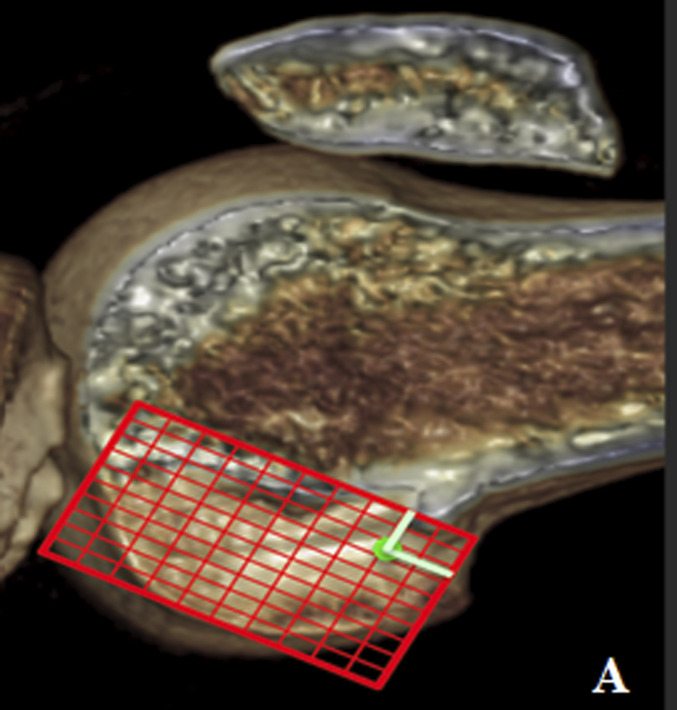

**Fig. 2-B:**
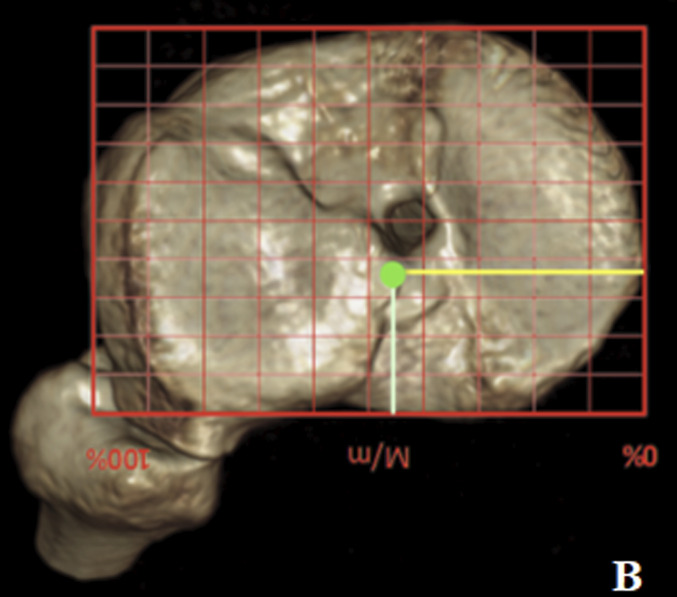

**Figs. 3-A and 3-B** Sagittal view (**Fig. 3-A**) and coronal view (**Fig. 3-B**) showing bony fragments of the posterior root of the MM.

**Fig. 3-A:**
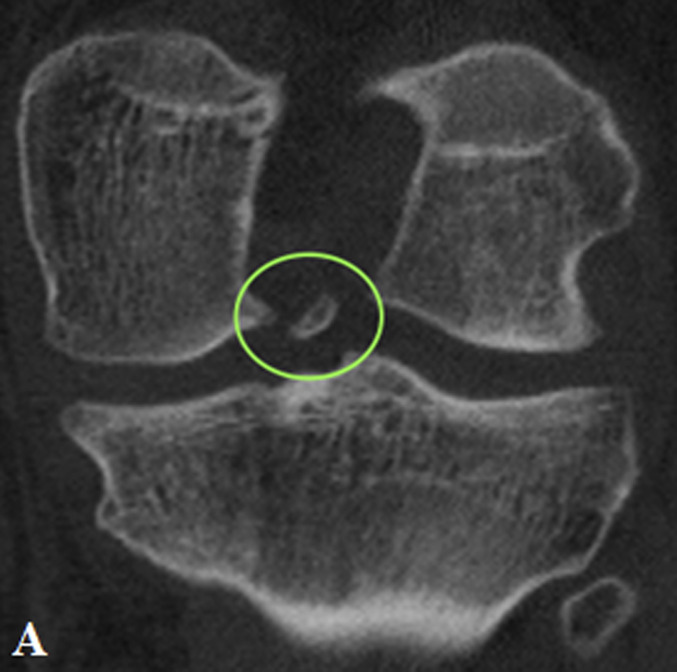

**Fig. 3-B:**
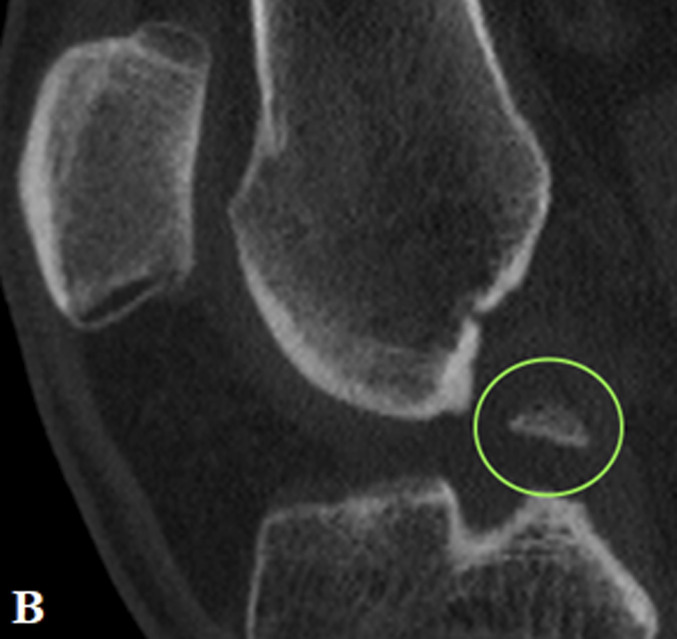

### Surgery

**Fig. 4:**
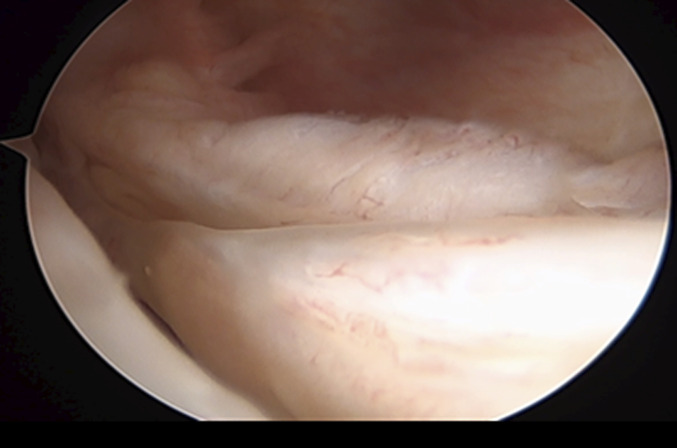
Direct visualization of the ramp lesion through the intercondylar notch view.

**Fig. 5:**
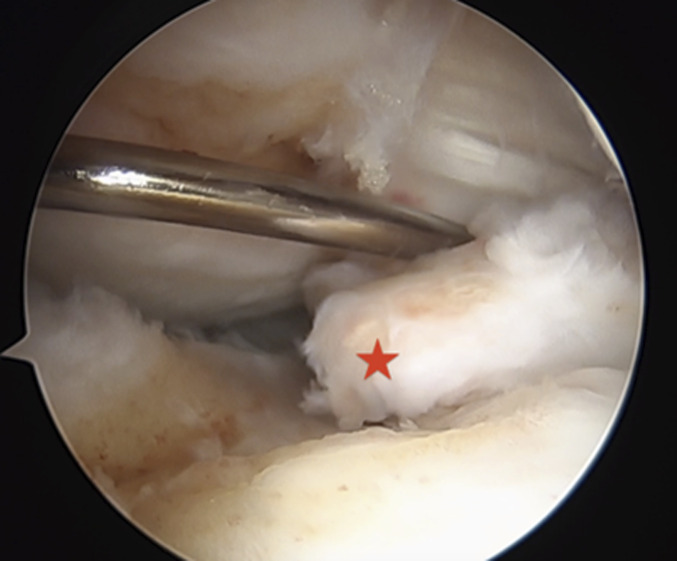
Direct visualization of the bony fragment (*) anterior to the Posterior Cruciate Ligaments.

Under spinal anesthesia and after antibiotic prophylaxis, the patient was placed supine with the tourniquet at the proximal thigh. The surgery began with ipsilateral patellar bone-tendon-bone autograft harvest and its preparation on a separate table. Knee arthroscopy was performed using standard anterolateral and anteromedial portals with a 30° arthroscope. On arthroscopic inspection through the intercondylar view, we confirmed a complete ramp lesion (Thaunat classification: type 4) and bony avulsion fracture of the posterior horn of the MM (LaPrade classification: type 5) (Figs. 4 and 5).

No remnants of the previous ACL graft were identified. The Posterior Cruciate Ligament appeared normal, and no other lesions were observed on the lateral compartment.

The ramp lesion was repaired using an all-inside technique with a dedicated hook. The first step was to recreate the anatomic attachment of the meniscus to the posterior capsule: a posteromedial (PM) portal was created, and using a 25° curved hook, a needle was passed through the medial meniscus and through the posterior capsule to a vertical suture. (Figs. 6-A and 6-B).

**Figs. 6-A and 6-B** 25° Curved hook used to repair the ramp lesion inserted through the PM portal.

**Fig. 6-A:**
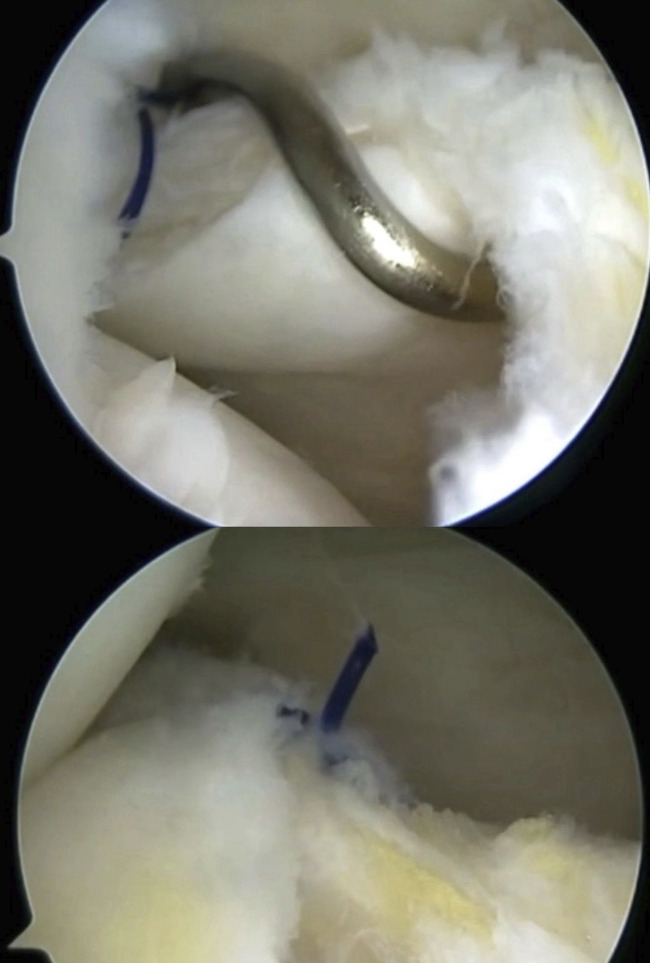

**Fig. 6-B:**
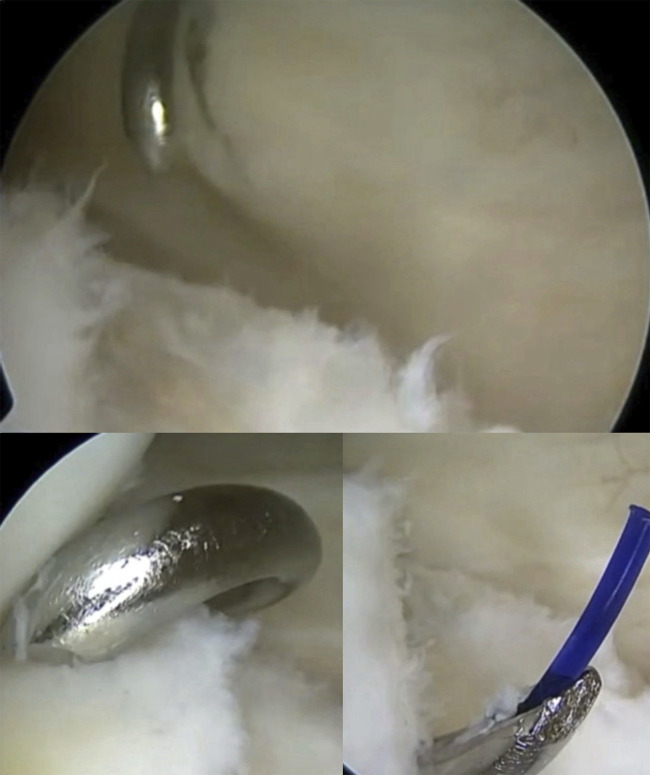

**Fig. 7:**
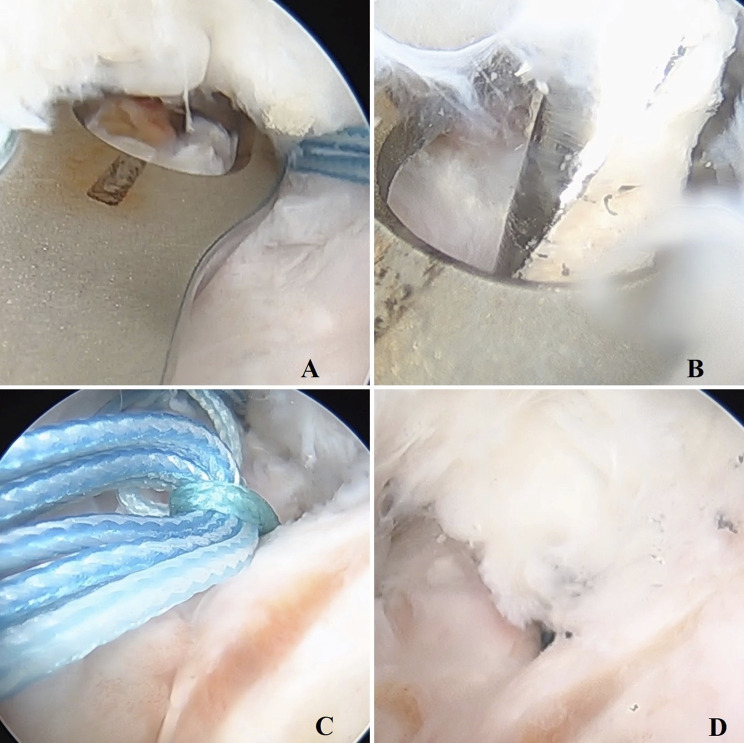
**Figs. 7-A through 7D** Posterior root of MM repair. (**Fig. 7-A**) Identification of the shiny white fibers using the tibial guide; (**Fig. 7-B**) K-wire used to create the tibial tunnel; (**Fig. 7-C**) suture retrieval through the tibial tunnel; and (**Fig. 7-D**) reduced root tear is confirmed.

Once the ramp lesion was sutured, a transtibial technique was used to address the root tear: Using a meniscus root self-retrieving suture device, 2 sutures were applied on the root of the meniscus and retrieved from the anteromedial portal. A tibial guide was then used to create the tibial tunnel with a 2.4-mm K-wire from the anterior tibial cortex to the anatomic insertion of the posterior root of the MM, using the shiny white fibers as posterior reference^9^. Then using a suture shuttling device, these sutures were retrieved through the tibial tunnel and then tied to the Attachable Button System (Arthrex) on the anterior tibial cortex (Fig. 7).

Subsequently, the ACL revision was performed by creating 2 new tunnels and using a patellar bone-tendon-bone autograft. The tibial tunnel was semianatomic, and the femoral tunnel was vertical-anterior in relation to the anatomic footprint. To avoid convergence of the meniscal and ACL tunnels, it was crucial to create a meniscal tunnel that is more horizontally inclined and with a lateral entry point. To further stabilize the ACL repair, anterolateral stabilization was performed using the technique described by Ellison et al. applying a knotless suture anchor on Gerdy's tubercle^4^.

### Postoperative Rehabilitation

The patient followed a non–weight-bearing protocol for 4 weeks to protect the meniscal sutures, while maintaining 0 to 90° knee range of motion. Starting from the fifth week, a progressive weight-bearing protocol was implemented, allowing for full weight-bearing by the eighth week. Clinical follow-ups were conducted at 1, 3, 6 months, and 1 year after surgery. The patient has completely recovered and returned to normal-high physical activity 1 year after surgery. There was a significant improvement in all subjective functional scores compared with presurgery measurements: The Lysholm score improved from 64 to 92, the Tegner activity scale increased from 2 to 9, the International Knee Documentation Committee score improved from 48.45 to 77.31, and the Western Ontario and McMaster University score was 100% after surgery (Figs. 8 and 9).

**Figs. 8-A and 8-B** Postoperative radiography in coronal (**Fig. 8-A**) and sagittal (**Fig. 8-B**) view.

**Fig. 8-A:**
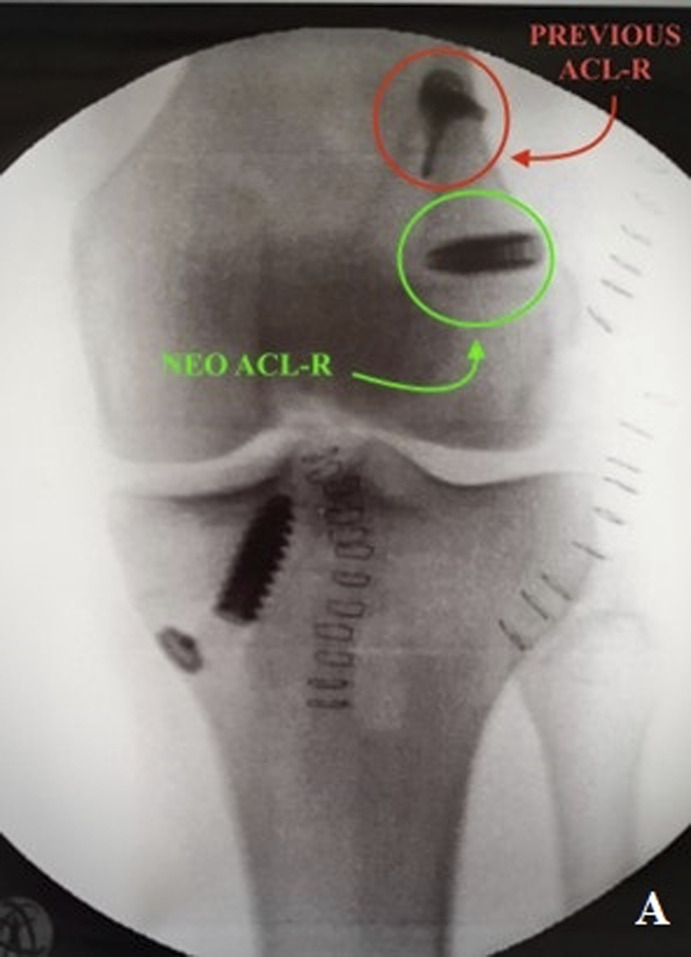

**Fig. 8-B:**
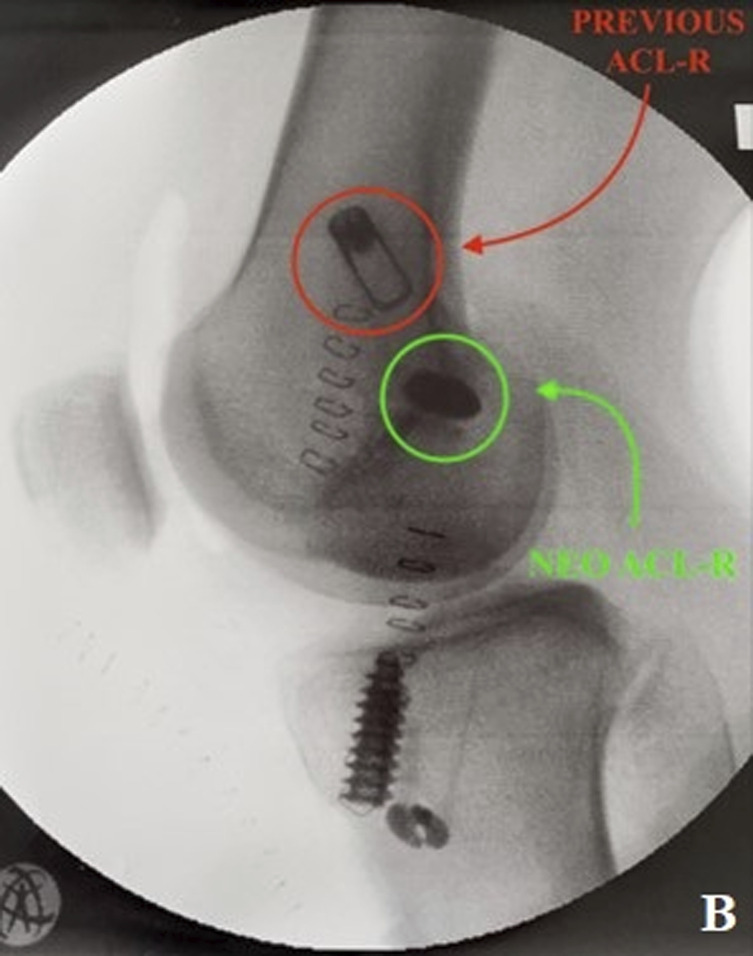

**Fig. 9-A**Thaunat classification of ramp lesions of the medial meniscus. **Fig. 9-B**LaPrade classification of meniscal root tears.

**Fig. 9-A:**
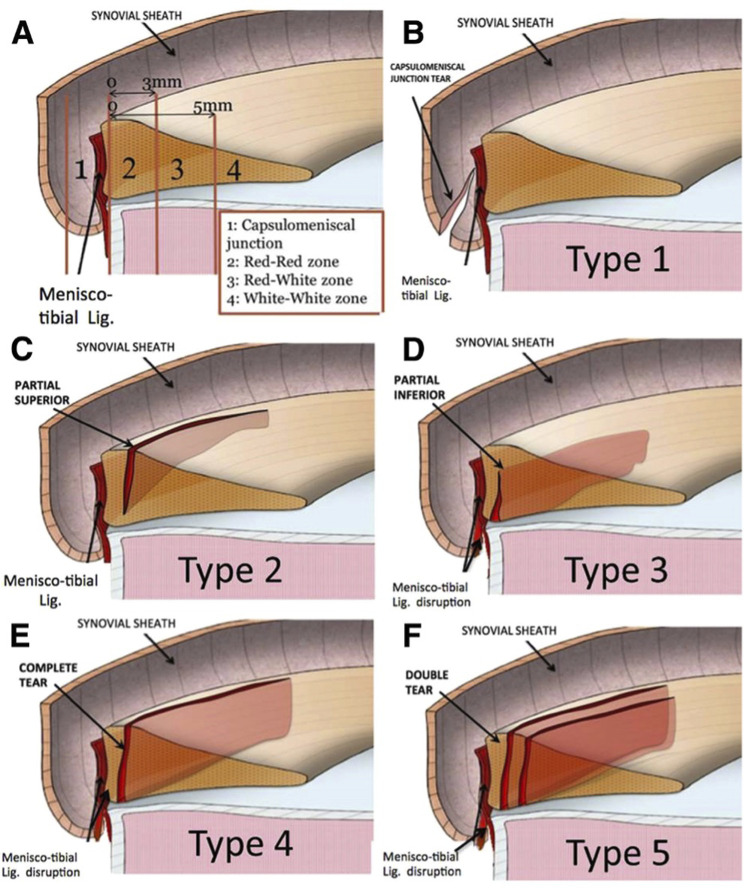

**Fig. 9-B:**
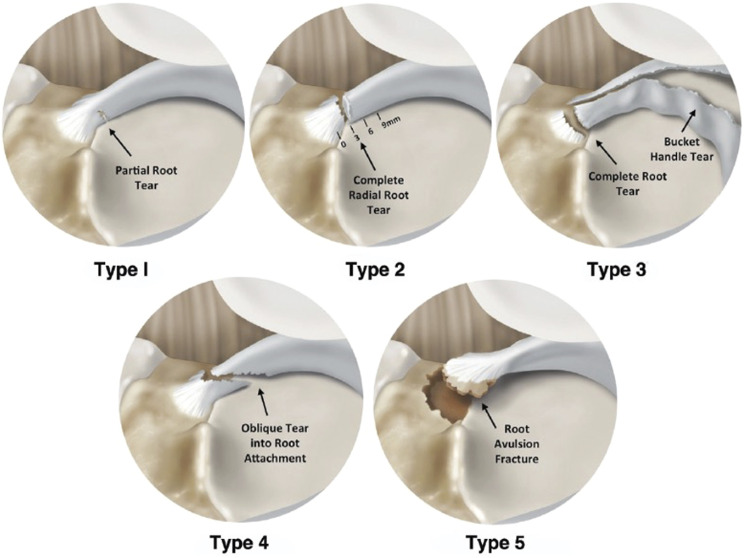

## Discussion

Ramp lesions combined with bony root avulsion affecting the same meniscus are not well reported in the scientific literature, especially with respect to their combined effect on the knee's biomechanics and their appropriate treatment.

Our case report highlights the possibility of occurrence of these lesions together associated with an ACL rupture and describes a potential treatment modality. Our patient underwent arthroscopic repair using an all-inside technique for the ramp lesion, a transtibial technique to address root lesions in addition to ACL reconstruction revision. The treatment showed promising short-term outcomes.

Ramp lesions and root tears are classified separately according to Thaunat and LaPrade classifications, respectively^1,2,8^. Ramp lesions are particularly difficult to identify and are often referred to as hidden lesions because the MRI’s sensitivity in detecting them ranges from 53.9 to 84.6%,^7^ and therefore, a thorough arthroscopic inspection through the intercondylar notch view is mandatory to rule them out. If these lesions are left untreated or misdiagnosed, they can have detrimental effects on the knee’s biomechanics especially in the context of an ACL reconstruction or revision as they increase the risk of graft failure because of joint instability^5^.

Our case highlights the possibility of a combined pattern involving root and ramp lesions within the same meniscus emphasizing the importance of a careful arthroscopic inspection. This specific meniscal injury pattern should be recognized and documented, potentially warranting consideration as a new addition to Thaunat and LaPrade classifications as type 6.

Surgeons must actively look for indirect signs of these lesions on MRI when addressing ACL rupture and be prepared to address them in case they are diagnosed intraoperatively during arthroscopy.

In summary, it is crucial to approach meniscal lesions with an open mind. The absence of a classification including the combination we presented does not negate its existence. Therefore, during arthroscopic ACL reconstructions, surgeons should exercise utmost caution and proactively search for any associated lesions.
